# Potential of *Akkermansia muciniphila* and its outer membrane proteins as therapeutic targets for neuropsychological diseases

**DOI:** 10.3389/fmicb.2023.1191445

**Published:** 2023-06-27

**Authors:** Fenghua Zhang, Dali Wang

**Affiliations:** ^1^Department of Laboratory Medicine, Shanghai University of Medicine and Health Sciences Affiliated Zhoupu Hospital, Shanghai, China; ^2^Center for Clinical and Translational Medicine, Shanghai University of Medicine and Health Sciences, Shanghai, China

**Keywords:** *Akkermansia muciniphila*, Amuc_1100, neuropsychological disease, probiotics, treatment

## Abstract

The gut microbiota varies dramatically among individuals, and changes over time within the same individual, due to diversities in genetic backgrounds, diet, nutrient supplementations and use of antibiotics. Up until now, studies on dysbiosis of microbiota have expanded to a wider range of diseases, with *Akkermansia muciniphila* at the cross spot of many of these diseases. *A. muciniphila* is a Gram-negative bacterium that produces short-chain fatty acids (SCFAs), and Amuc_1100 is one of its most highly expressed outer membrane proteins. This review aims to summarize current knowledge on correlations between *A. muciniphila* and involved neuropsychological diseases published in the last decade, with a focus on the potential of this bacterium and its outer membrane proteins as therapeutic targets for these diseases, on the basis of evidence accumulated from animal and clinical studies, as well as mechanisms of action from peripheral to central nervous system (CNS).

## Introduction

The human gut hosts around 10^13^–10^14^ microorganisms from different species that compete, suppress and collaborate with each other to achieve delicate functions. During past decades, the knowledge on gut microbiota expanded from being merely a colonizer to being a keeper of a stable microenvironment. Importantly, these highly diverse intestinal microorganisms participated not only in maintenance of a healthy gut, but also in pathogenesis and progression of many diseases where dysbiosis occurs, via various mechanisms (Mosca et al., 2016; Hughes et al., 2018; Chen et al., 2019; Laniewski et al., 2020; Wang et al., 2021; Geng et al., 2022). Though each disease presents a unique disturbance of gut microbiota, some species appear to be involved across multiple diseases, and therefore have become targets of interest, as they may either provide an insight to a common pathogenic pathway (Makdissi et al., 2023), or hold the potential to be used as a marker or target for diagnosis or treatment of these diseases (Mamic et al., 2023). The Gram-negative anaerobe *Akkermansia muciniphila*, which belongs to Verrucomicrobia phylum, is one of such species that has been shown to be dysregulated in an increasing number of diseases such as metabolic disorders (Xu et al., 2020) and intestinal diseases (Liu et al., 2022).

The best-studied strain of *A. muciniphila*, MucT (ATCC BAA-835), was first isolated from human feces in 2004 (Derrien et al., 2004). The bacterium colonizes at the mucosal interface between the oxic intestinal epithelium and the anoxic lumen throughout the human gut (Ouwerkerk et al., 2016), and constitutes up to 5% of total bacteria in human gastrointestinal tract under basal conditions (Bellenger et al., 2019). The 16S rRNA gene sequencing has revealed that *A. muciniphila* colonizes early in life, develops within 1 year to a level close to that in adults, and decreases with aging (Collado et al., 2007). Since the discovery of this bacterium, numerous studies have shown its favorable role in metabolism (Plovier et al., 2017; Depommier et al., 2019; Rao et al., 2021; Daniel et al., 2023) and anti-inflammation (Grander et al., 2018; Hanninen et al., 2018; Ansaldo et al., 2019). Specifically, *A. muciniphila* has been consistently shown to be present with a relatively higher abundance in clinically healthy and lean individuals, compared with overweight or obese individuals (Derrien et al., 2017), with a higher abundance being correlated with a higher level of metagenome diversity (Le Chatelier et al., 2013). Importantly, *A. muciniphila* tends to co-exist with bacteria typically associated with a healthy human gut, such as *F. prausnitzii*, *Gordonibacter* spp. and *Methanobrevibacter smithii* (Dao et al., 2016), but not with those associated with unhealthy gut such as *Prevotella* spp. (Arumugam et al., 2011). Therefore, *A. muciniphila* has been proposed as a next-generation probiotic (Zhai et al., 2019). In fact, the direct administration of live (Depommier et al., 2019) or pasteurized bacterium grown on a synthetic medium (Plovier et al., 2017) were proved safe in humans. Notably, the pasteurized *A. muciniphila* has been approved as novel food by EFSA (Efsa Panel on Nutrition, N. F et al., 2021). *A. muciniphila* was detected elevated in abundance in athletes gut microbiome (Petersen et al., 2017), and could be upregulated by probiotics supplementation (Huang et al., 2020). However, contradictory results with a lower abundance of *A. muciniphila* were found in the rugby players and cyclists compared to controls who were not sedentary but exercised normally (Hintikka et al., 2022).

Though mainly studied in metabolic and gastrointestinal disorders previously, analysis of this species has recently been extended to neuropsychological diseases. Most of these studies have revealed a change in its relative abundance in both patients and disease models, which brings to the following questions: (i) whether the bacterium plays a unique role in pathogenesis of diseases; (ii) whether it serves as a biomarker to predict trends towards certain diseases and/or disease progression; (iii) whether modulation of this species, its outer membrane proteins or its metabolites would potentially be beneficial in the long-term management of these diseases. To answer these questions, this narrative review summarizes the correlations between *A. muciniphila* and neuropsychological diseases by reviewing the major findings from human and animal studies published in the last decade. Additionally, the potential therapeutic values and modulating strategies for *A. muciniphila* and underlying mechanisms are also discussed.

### *Akkermansia muciniphila* and gut barrier

The adhesion study has revealed a more significant binding of *A. muciniphila* to human extracellular matrix protein laminin compared with its binding to bovine serum albumin (BSA; Reunanen et al., 2015). The adhesion level of *A. muciniphila* to intestinal epithelium and mucus was as low as <1% (Reunanen et al., 2015), and its absence of mucus-binding domain showcased in a comprehensive analysis of its genome was consistent to its mucinolytic nature (van Passel et al., 2011). Moreover, *A. muciniphila* was shown to bind to human enterocytes at a comparable level to the positive-control, and its binding site was expressed on epithelial cell surface at all differentiation stages (Reunanen et al., 2015). Therefore, *A. muciniphila* may participate in the competitive exclusion of pathogenic microorganism from sites of damage.

**Figure 1:**
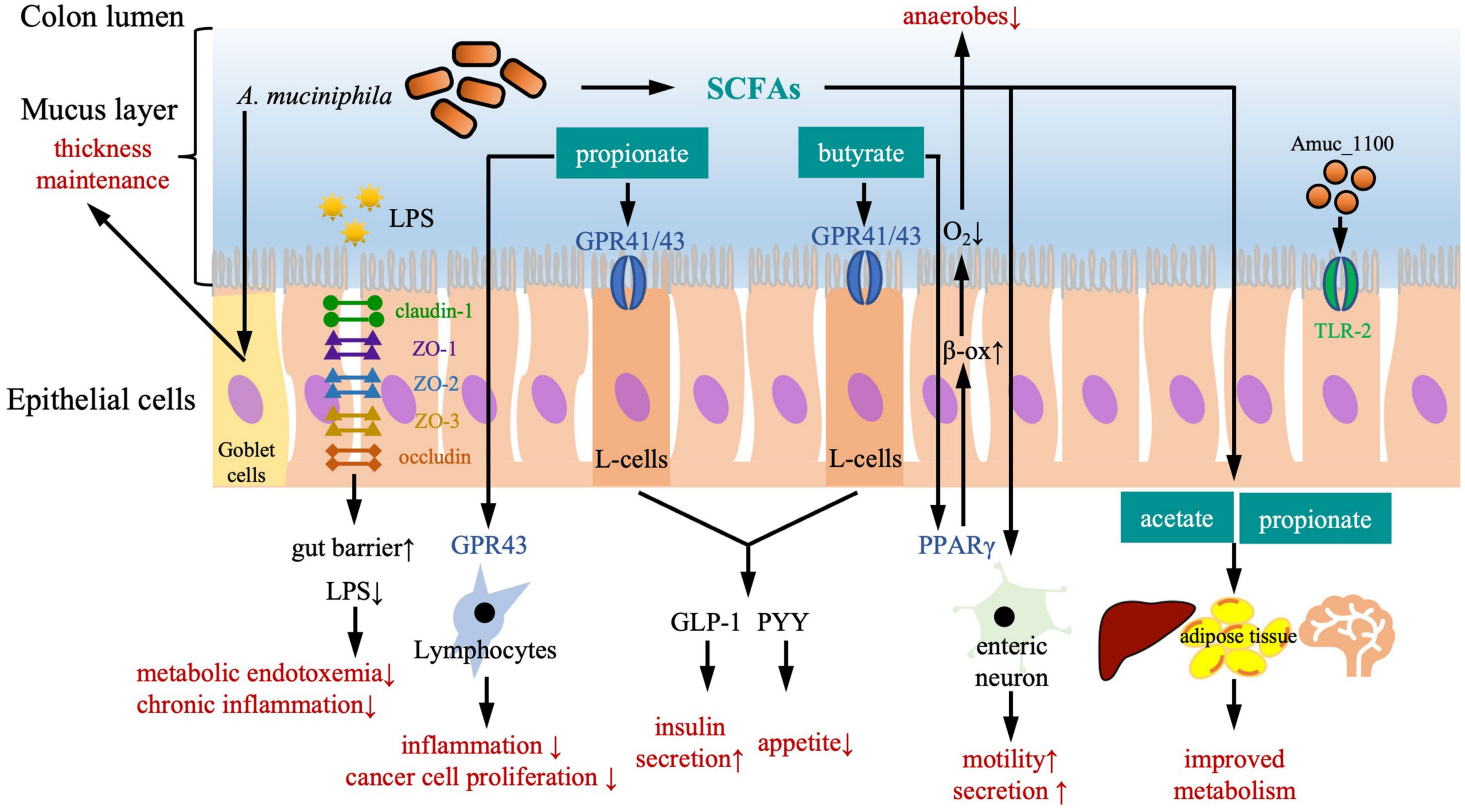
The protective effects of *Akkermansia muciniphila* against diseases. Biochemically, *A. muciniphila* exerts its protective function in a SCFA-dependent manner. Both butyrate and propionate bind G protein coupled receptor (GPR)-43 and GPR-41 that are expressed on the enteroendocrine L-cells, subsequently trigger secretion of gut peptides such as glucagon-like peptide-1 (GLP-1) and peptide YY (PYY), leading to a reduction of food intake and an improvement of glucose metabolism. Propionate also acts as an immune regulator by binding to GPR-43, which is expressed on lymphocytes to reduce inflammation and maintain an appropriate immune defense, as well as to reduce proliferation of cancer cells. Butyrate activates peroxisome proliferator-activated receptor-γ (PPAR-γ), leading to beta-oxidation and oxygen consumption, and maintains anaerobic condition in the gut lumen. In addition, SCFAs also trigger secretory activity and motility in intestine via stimulating enteric neuron signaling. Effects on remote organs are modulated by acetate and propionate in the circulation, leading to an improved metabolism of liver, adipose tissue and brain. Physically, *A. muciniphila* enhances the gut barrier to defend intrusion of harmful particles and pathogens in several ways. Firstly, *A. muciniphil* upregulates expression of tight-junction proteins including claudin-1, zonula occluden (ZO)-1, ZO-2, ZO-3, and occludin, improving gut barrier and reducing gut permeability for LPS. Secondly, the pili-like membrane protein (Amuc_1100) from *A. muciniphila* is also involved in maintaining host immunological homeostasis by activating toll-like receptor 2 (TLR-2) and improving gut barrier function. Thirdly, *A. muciniphila* acts on Goblet cells to stimulate the secretion of mucus and thereby maintain the thickness of mucus layer.

*A. muciniphila* plays an essential role in maintaining gut barrier and mucus layer integrity (Figure 1). On one hand, it facilitates mucus production (Collado et al., 2007). Thus, the protective effects from accumulation of this bacterium could be associated with thickness of the mucus layer, reduction of gut permeability, and prevention from leakage of bacterial lipopolysaccharide (LPS) into the bloodstream (Everard et al., 2013; Zhai et al., 2019). On the other hand, a thin mucus layer, often seen in diseases like inflammatory bowel disease (IBD), is considered harmful by exposing the gut mucosa to microbial antigens and leading to a more permeable barrier for endogenous proteins and endotoxin. Notably, a low abundance of *A. muciniphila* in the gut is often detected under these pathological conditions (Zhang et al., 2020).

### *Akkermansia muciniphila* as an SCFAs-producing bacterium

The short-chain fatty acids (SCFAs) are generated from fermentation of dietary fibers by intestinal bacteria and are mainly composed of butyrate, propionate, and acetate. *A. muciniphila* mainly produces propionate and acetate, while butyrate is produced by its neighbouring symbionts (Ottman et al., 2017a). SCFAs have several beneficial effects including maintaining epithelial barrier function, diminishing oxidative DNA damage, regulating cytokine production, promoting anti-inflammation, and stimulating immune functions (Maslowski and Mackay, 2011) and metabolic health (Muller et al., 2019). The majority of butyrate is utilized in the gut, which has been shown to increase energy expenditure by promoting fat oxidation and activating brown adipose tissue (Roshanravan et al., 2017), as well as to increase *A. muciniphila* levels (Arora et al., 2019). Meanwhile, a large amount of acetate and a small proportion of propionate reach the circulation and improve the metabolism of liver, adipose tissue and brain (Gomes et al., 2018). Notably, *A. muciniphila* failed to produce propionate in the absence of vitamin B12, which is a cofactor for converting succinate to propionate via methylmalonyl-CoA synthase (Karcher et al., 2021).

As one of the key SCFAs-producing bacteria, *A. muciniphila* has been hypothesized to act as a protective player against a variety of central and peripheral diseases in a SCFAs-dependent manner (Cani, 2018; Caesar, 2019). The administration of this species may lead to functional symbiosis, which results in a status rich of the SCFAs. For instance, the oral administration of an *A. muciniphila* subtype, which produces SCFAs including acetic acid, propionic acid and isovaleric acid, has been shown to exert beneficial effects against body weight gain, hyperglycosemia and cognitive impairment in HFD-treated mice (Wu et al., 2020).

### *Akkermansia muciniphila* and neurovascular integrity

Under pathological conditions, a variety of inflammatory stimuli, such as cytokines, LPS, amyloid β (Aβ) and tau fragments may initiate an inflammatory response in endothelial cells of the blood brain barrier (BBB), which activates a variety of signaling pathways and consequently results in neurovascular abnormalities, destruction of the BBB and activation of peripheral paracrine cells (Montagne et al., 2017). Accumulating evidence has shown a critical role of gut microbiota in regulation of neurovascular integrity. For instance, in germ-free mice, the lack of butyric acid due to deficiency of SCFAs-producing flora has led to a significant increase in BBB permeability (Braniste et al., 2014). As one of the key SCFAs-producing bacteria, the lack of *A. muciniphila* caused a damage to the tight junction (TJ) of the BBB and the intestinal barrier (Li et al., 2016). On the contrary, the upregulation of *A. muciniphila* following ketogenic diets (KD) in mice could lead to an increase in cerebral blood flow and transport of P-glycoprotein through the BBB to facilitate the clearance of Aβ (Ma et al., 2018). Therefore, interventions modulating *A. muciniphila* at the early stage of cognitive impairment may reduce the risk of developing Alzheimer’s disease (AD) by improving neurovascular functions.

### The outer membrane protein

Amuc_1100, which is a 32 kDa pili-like protein composed of four ɑ helixes and a four-strand antiparallel β fold, is one of the most expressed outer membrane proteins of *A. muciniphila* (Ottman et al., 2017a). It remains stable at different temperatures and after pasteurization (Plovier et al., 2017), playing a key role in maintaining host immunological homeostasis and improving gut barrier function (Plovier et al., 2017; Ottman et al., 2017a). Specifically, the interaction between Amuc_1100 and mucin is required for the colonization of the bacterium (Shin et al., 2019). Actually, *A. muciniphila* could regulate the expression of TJ proteins (Occludin, zonula occluden (ZO-1) and Claudin-1) via activation of 5’AMP-activated protein kinase (AMPK) pathway by *A. muciniphila*-derived extracellular vesicles (AmEVs) as well as activation of Toll-like receptor 2 (TLR2) by Amuc_1100 (Ottman et al., 2017b; Chelakkot et al., 2018; Ashrafian et al., 2019). Amuc_1100 has been reported to interact with TLR2, directly and subsequently promote the expression of serotonin (5-HT) synthesis rate-limiting enzyme tryptophan hydroxylase 1 (Tph1) in Rin-14B cells and reduced the expression of the serotonin reuptake transporter (SERT) in Caco-2 cells, thereby improve the biosynthesis and extracellular availability of 5-HT (Wang et al., 2021). Recent studies in murine models have shown that the protective effects of *A. muciniphila* against diet-induced obesity was partially due to the interaction between Amuc_1100 and TLR2/TLR4 (Plovier et al., 2017), subsequently facilitating the production of cytokines [e.g., interleukin (IL)-6, IL-8, IL-10] (Ottman et al., 2017b) and upregulating TJ proteins (Plovier et al., 2017; Ashrafian et al., 2019). Recent studies have also suggested that Amuc_1100 may play an important role in regulating host amino acid metabolism. In a mouse model of colitis, Amuc_1100 exerted beneficial effects by reducing infiltrating macrophages and CD8+ cytotoxic T lymphocytes in the colon, and by enhancing indoleacetic acid (IAA) and indoleacrylic acid (IA) levels in the microbial tryptophan (Trp) metabolic pathway to activate aryl hydrocarbon receptor (AhR) signaling (Wang et al., 2020). In addition, Amuc_1100 could also inhibit kynurenine (Kyn) pathway by up-regulating Kyn level, down-regulating 2-picolinic acid (PIC) level, and affecting PIC/Kyn ratio (Gu et al., 2021).

### *Akkermansia muciniphila* in neuropsychological diseases

**Figure 2:**
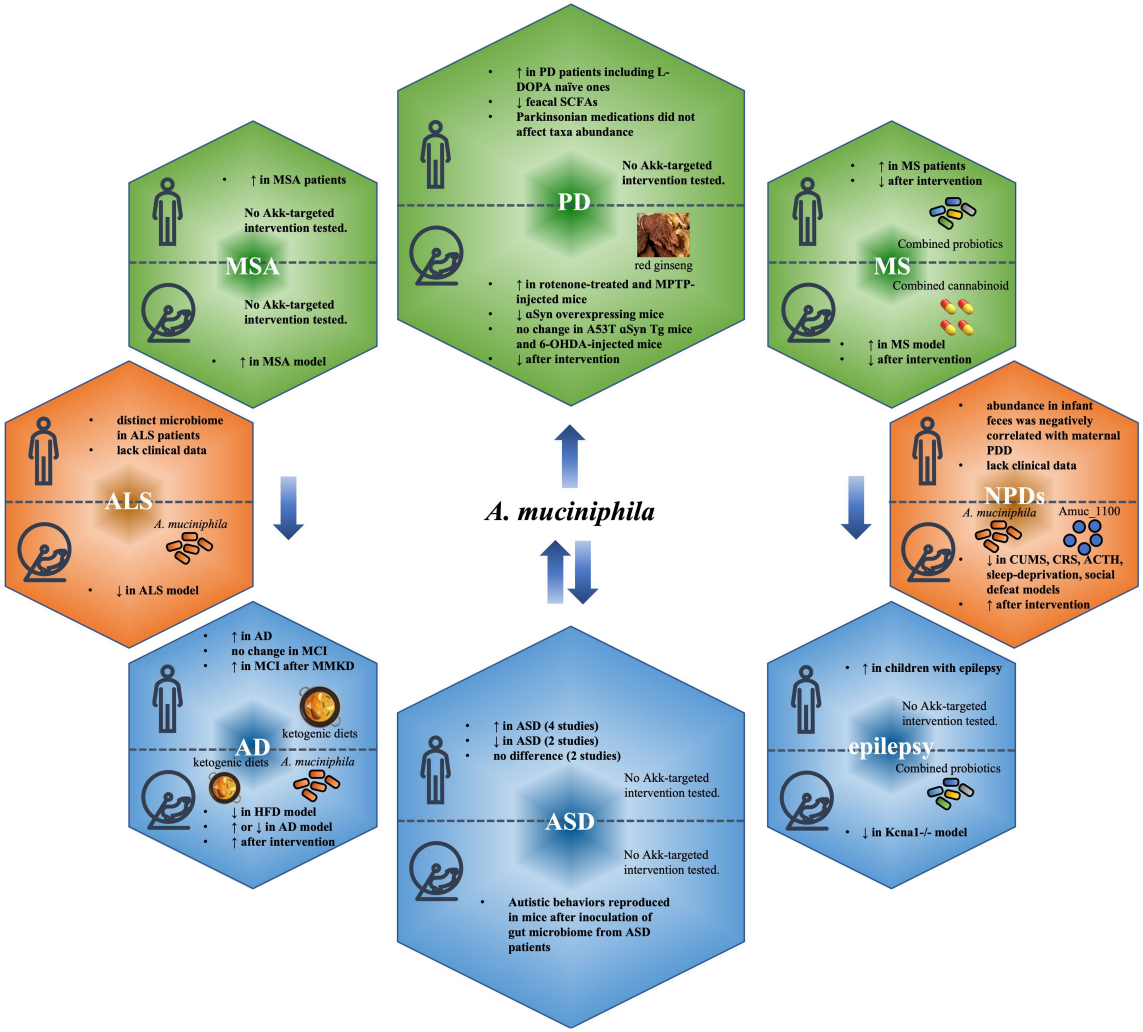
Change of *Akkermansia muciniphila* abundance in neuropsychological diseases and attempts of modulation of *A. muciniphila*. Overall, the abundance of *A. muciniphila* was shown to decrease (red boxes) significantly in amyotrophic lateral sclerosis (ALS) and neuropsychiatric disorders (NPDs), increase (green boxes) significantly in multiple system atrophy (MSA), multiple sclerosis (MS) and Parkinson’s disease (PD), but remains ambiguous (blue boxes) in Alzheimer’s disease (AD) and cognitive deficits, autism spectrum disorder (ASD) and epilepsy.

Raised since about a decade ago, the concept of ‘microbiota-gut-brain axis’, which provides bidirectional communication pathways between the gut and the brain (Cryan and O'Mahony, 2011; Sherwin et al., 2018), has linked dysbiosis of intestinal microbiota to pathogenesis of diseases of the central nervous system (CNS). This brings new insights into the understanding of disease mechanisms, and the development of biomarkers and potential treatments of these debilitating diseases. Overall, the abundance of *A. muciniphila* was shown to decrease significantly in amyotrophic lateral sclerosis (ALS; Blacher et al., 2019) and neuropsychiatric disorders (NPDs; McGaughey et al., 2019; Song et al., 2019; Aatsinki et al., 2020; Park et al., 2020; Cheng et al., 2021; Ding et al., 2021), increase significantly in Multiple System Atrophy (MSA; Derrien et al., 2011; Wan et al., 2019), Multiple Sclerosis (MS; Cantarel et al., 2015; Jangi et al., 2016; Berer et al., 2017; Cekanaviciute et al., 2017, 2018; Tankou et al., 2018; Al-Ghezi et al., 2019; Probstel et al., 2020) and Parkinson’s disease (PD; Braak et al., 2006; Holmqvist et al., 2014; Keshavarzian et al., 2015; Remely et al., 2015; Sampson et al., 2016; Unger et al., 2016; Bedarf et al., 2017; Hill-Burns et al., 2017; Heintz-Buschart et al., 2018; Gorecki et al., 2019; Hertel et al., 2019; Li et al., 2019; Cirstea et al., 2020; Dodiya et al., 2020; Nishiwaki et al., 2020; Pietrucci et al., 2020; Vidal-Martinez et al., 2020; Hou et al., 2021; Jeon et al., 2021; Augustin et al., 2023), but remains ambiguous in Alzheimer’s disease (AD) and cognitive deficits (Harach et al., 2017; Vogt et al., 2017; Ma et al., 2018; Nagpal et al., 2019; Yang et al., 2019; Chen et al., 2020), autism spectrum disorder (ASD; Wang et al., 2011; De Angelis et al., 2013; Kang et al., 2013; Inoue et al., 2016; Lee et al., 2017; Strati et al., 2017; Sharon et al., 2019; Zou et al., 2020; Zurita et al., 2020) and epilepsy (Olson et al., 2018; Huang et al., 2019; Figure 2).

#### Amyotrophic lateral sclerosis

*A. muciniphila* is negatively associated with ALS, which is indicated by a gradually decreased abundance of *A. muciniphila* in ALS mice (Blacher et al., 2019). The same research group identified a distinct microbiome and metabolite configurations in a small cohort study comparing ALS patients with household controls (Blacher et al., 2019). However, larger scaled investigations are required to confirm such alterations in microbiome and the effectiveness of Akk-targeted interventions.

#### Neuropsychiatric disorders

A decreased abundance of *Akkermansia* has been observed in multiple depressive-like animal models (Table 1), including (i) chronic unpredictable mild stress (CUMS)-induced or chronic restraint stress (CRS)-induced mouse models (Cheng et al., 2021; Ding et al., 2021); (ii) a chronic adrenocorticotrophic hormone (ACTH)-induced depression rat model (Song et al., 2019); (iii) a water-stress-induced sleep-deprived mouse model (Park et al., 2020); (iv) a mouse model exhibiting depressive- and anxiety-like behavior following social defeat (McGaughey et al., 2019). The abundance of *A. muciniphila* was negatively correlated with anxiety- and depressive-like behaviors, which were indicated by sucrose preference test and open-field test (McGaughey et al., 2019).

**Table 1:** Association between *Akkermansia muciniphila* and representative neurological diseases in disease models.

Groups	*Akkermansia muciniphila* level	Outcome	Intervention	Ref.
**AD and cognitive deficits**				
Young healthy mice (12–14-week-old)	↑After intervention	↑Clearance of Aβ	16-Week ketogenic diet	Ma et al. (2018)
APP/PS1 WT Germ free APP/PS1 (*n* = 6–8/group)	↓In APP/PS1 mice	Negative correlation between *A. muciniphila* and Aβ42	None	Harach et al. (2017)
APP/PS1 WT (*n* = 14–24 for 1-, 2-, 3-, 9-month old group; *n* = 31–34 for 6-month-old group)	↑In APP/PS1 mice at 2-,6-,9- month old	Aβ plaques in the cortex did not show up until 3-month old Alteration of gut microbiome profile occurs before amyloidosis and microglial activation in AD mice	None	Chen et al. (2020)
APP/PS1 on HFD APP/PS1 on normal chow diet WT (*n* = 10/group for APP/PS1, *n* = 6/group for WT)	/	Improved glucose and lipid profile ↑Clearance of Aβ40-42 in cortex Improved completion rate in Y-maze test	6-Month Akk by gavage	Ou et al. (2020)
AD-like rats with periodontitis WT (*n* = 4-5/group)	↑After intervention	↑Clearance of Aβ Improved cognition	6-Month Akk by oral administration	He et al. (2022)
C57BL/6 mice with HFD C57BL/6 mice with normal diet	/	Body weight and glucose control Improved spatial memory	10-Month Akk by gavage	Wu et al. (2020)
C57BL/6 mice with HFD C57BL/6 mice with normal diet (*n* = 8/group)	↓Post HFD	Improved gut permeability ↓Hippocampal microgliosis & proinflammatory cytokines Improved contextual/spatial learning & memory	28-Day Akk (ATCC BAA845) by gavage	Yang et al. (2019)
SD rats with HFHC SD rats with normal diet	↑After intervention	Reversed HFHC-induced cognitive dysfunction (spatial working memory & novel object recognition)	28-Day Akk (CIP107961) by gavage	Higarza et al. (2021)
**ALS**				
Sod1-Tg mice (germ free) Sod1-Tg mice (antibiotic treated)	↓in Sod1-Tg mice	Accumulated nicotinamide in the CNS Mitigate disease progression	Akk (MucT & ATCC BAA-2869) supplementation	Blacher et al. (2019)
**NPDs**				
CUMS mouse model No CUMS control	↓Post CUMS	↑5-HT ↑BDNF Improved CUMS-induced behavior disorder	Amuc_1100 by oral administration	Cheng et al. (2021)
CRS mouse model No CRS control	↓Post CRS	Ameliorated depressive-like behavior Restored corticosterone, dopamine, BDNF	3-Week Akk by gavage	Ding et al. (2021)
ACTH-induced rat model No injection control	↓Post ACTH chronic injection	Depressive-like behavior	None	Song et al. (2019)
Water stress and sleep-deprived mouse model No water stress and sleep deprivation controls (*n* = 6/group)	↓Post water stress and sleep deprivation ↑After intervention	Improved depressive-like behavior	Melatonin injection	Park et al. (2020)
Social defeat mouse model (*n* = 20) No social defeat controls (*n* = 19)	↓Post social defeat	Depressive- and anxiety-like behaviors	None	McGaughey et al. (2019)
Alcohol-LPS mouse model Control mice	↑After intervention	Ameliorated depressive-behavior ↓LPS, neuroinflammation	Akk supplementation	Guo et al. (2022)
**MSA**				
Tg CNP-aSyn MSA mouse model WT (*n* = 5/group)	↑In MSA mice	None	None	Vidal-Martinez et al. (2020)
**MS**				
Monocolonized GF mice Specific pathogen-free mice (*n* = 3–8/group)	↑In MS mice	None	None	Cekanaviciute et al. (2017)
EAE mice Specific pathogen-free mice (*n* = 3–8/group)	↑In EAE mice ↓After intervention	Attenuated EAE ↓Proinflammatory cytokines (IL-17, IFN-ɣ) ↑Anti-inflammatory cytokines (IL-10, TGF-β)	Combined cannabinoid treatment (THC + CBD at 1:1 ratio)	Al-Ghezi et al. (2019)
**PD**				
Rotenone-treated mice WT (*n* = 4–6/group)	↑In rotenone-treated mice	Intestinal hyperpermeability Endotoxemia Tight junction barrier loss	None	Dodiya et al. (2020)
MPTP-injected mice Control	↑In MPTP-injected mice ↓After intervention	Prevented MPTP-induced behavioral impairments Prevented neurodegenerative damages	Korean red ginseng by oral administration	Jeon et al. (2021)
A53T ɑSyn Tg mice WT (*n* = 5/group)	No change	None	None	Vidal-Martinez et al. (2020)
6-OHDA-injected mice Control (*n* = 6–12/group)	No change	None	None	Hou et al. (2021)
Thy1-ɑSyn mice WT	↓In PD mice	None	None	Gorecki et al. (2019)
**Epilepsy**				
Kcna1−/− mice WT	↑After intervention	Protected against epilepsy	3-Week combined probiotic supplementation	Olson et al. (2018)

ACTH, adrenocorticotrophic hormone; AD, Alzheimer’s disease; ALS, amyotrophic lateral sclerosis; BDNF, brain-derived neurotrophic factor; CBD, cannabidiol; CRS, chronic restraint stress; CUMS, chronic unpredictable mild stress; HFD, high-fat diet; HFHC, high fat, high-cholesterol diet; KD, ketogenic diet; MS, multiple sclerosis; MSA, multiple system atrophy; THC, delta-9-tetrahydrocannabinol.

In human studies, the abundance of *A. muciniphila* in the fecal samples of 2.5 month-old infants was found negatively correlated with the symptoms of maternal prenatal psychological distress (PPD) in the FinnBrain Birth cohort study involving 398 mothers and their infants, suggesting that chronic PPD may affect the relative abundance of the species in the offsprings, which might subsequently contribute to changes in development outcomes (Aatsinki et al., 2020).

#### Multiple system atrophy

MSA is a sporadic, adult-onset, progressive neurodegenerative disease characterized by combinations of parkinsonian features, cerebellar ataxia, autonomic failure and pyramidal features. By metagenomic sequencing in feces of 15 MSA patients and 15 healthy controls, it showed an increase in genus *Akkermansia* and a decrease in genera *Megamonas*, *Bifildobacterium*, *Blauta* and *Agregatibacter* (Wan et al., 2019). The mechanisms behind an elevated level of *Akkermansia* in MSA remained unclear. Although *Akkermansia* was reported to be capable of upregulating genes involved in antigen presentation pathways, B and T cell receptor signaling, IL-4 signaling, as well as complement and coagulation cascades, it was suggested to be in pursuit of immune tolerance toward the bacteria and metabolic homeostasis of the colonized intestine (Derrien et al., 2011). Therefore, the higher abundance of this species in neurodegenerative diseases is possibly due to the tentative of the body to restore immune homeostasis, which is required to be further investigated.

#### Multiple sclerosis

MS is an autoimmune disease of the CNS characterized by demyelination, axonal damage and progressive neurologic disability. *A. muciniphila* abundance was positively associated with MS by increasing IFNɣ+ Th1 lymphocyte differentiation *in vitro*, indicating the crucial role of this bacterium in the pathogenesis of MS mediated via pro-inflammatory responses (Cekanaviciute et al., 2017). *A. muciniphila* has been shown in multiple case-controlled, cross-sectional and observational studies to increase in abundance in association with induction of pro-inflammatory responses in MS patients, compared with healthy controls (Jangi et al., 2016; Berer et al., 2017; Cekanaviciute et al., 2017, 2018; Tankou et al., 2018; Al-Ghezi et al., 2019; Probstel et al., 2020), whereas downregulation of *A. muciniphila* in MS has induced an anti-inflammatory immune response in the peripheral immune system, providing a potential target for MS treatment (Tankou et al., 2018; Table 2). Notably, *A. muciniphila* was positively associated with MS mixed cohorts consisted of untreated and under-therapy patients during disease remission, and the association between *A. muciniphila* and MS remained significant after correction for therapy (Jangi et al., 2016). In a recently published large-scaled clinical research (iMSMS Consortium. Electronic address: sergio.baranzini@ucsf.edu; iMSMS Consortium, 2022) on 576 MS patients and their household healthy controls, it was demonstrated a total of 16 species including *A. muciniphila* significantly elevated in untreated MS patients. Additionally, in the same study, *A. muciniphila* was also found to be significantly more represented in untreated relapsing–remitting and progressive MS patients, and but showed no difference in groups with disease modifying therapies. When transferring intestinal microbiota from MS twins to a spontaneous brain autoimmunity mouse model via fecal transplantation, microbiota from MS patients induced a significantly higher incidence of autoimmunity compared to that from their healthy co-twins (Berer et al., 2017). Unlike diabetes, disease-modifying therapies for MS have not altered the abundance of this species, compared to untreated patients (Jangi et al., 2016). In clinical practice, MS patients are often treated with glatiramer acetate (GA) and supplemented with vitamin D. Intriguingly, in a small pilot study, researchers have detected a higher enrichment of *A. muciniphila* in untreated MS patients after vitamin D supplementation, compared to GA-treated subjects and healthy controls (Cantarel et al., 2015), which may be indicative to the decision-making of therapeutic strategies for MS. By looking at immune transcriptional profiles in T cells and monocytes from MS patients, *A. muciniphila* was positively correlated with several genes from T-cells (including CASP1, TRAF5 and STAT5B) and with genes from monocytes (including MAPK14, MAPK1, LTBR, STAT5B, CASP1 and HLA-DRB1; Jangi et al., 2016).

**Table 2:** Association between *Akkermansia muciniphila* and representative neuropsychological diseases in humans.

Participants	*Akkermansia muciniphila* level	Outcome	Intervention	Ref.
AD and cognitive deficits				
AD (*n* = 25) Control (*n* = 25)	↑In AD	Negative correlation between bacterial abundance and Aβ42/ Aβ40 Positive correlation between bacterial abundance and p-Tau/p-Tau /Aβ42 Positive correlation between Bacteroides and YKL-40	None	Vogt et al. (2017)
Subjective memory complaints (*n* = 11) MCI (*n* = 9)	/	MMKD was associated with increased Aβ42 and decreased tau Improved performance on FCSRT	6-Week MMKD 6-Week AHAD	Neth et al. (2020)
MCI (*n* = 11) Control (*n* = 6)	No difference at baseline ↑On MMKD but not on AHAD	Improved AD biomarkers in CSF (Aβ42, Aβ40, total tau, p-tau 181)	6-Week MMKD 6-Week AHAD	Nagpal et al. (2019)
**NPDs**				
PDD (*n* = 398)	↓In infants of maternal PDD mothers	None	None	Aatsinki et al. (2020)
**MSA**				
MSA (*n* = 15) Healthy control (*n* = 15)	↑In MSA	None	None	Wan et al. (2019)
**MS**				
MS (*n* = 71) Healthy control (*n* = 71)	↑In MS	None	None	Cekanaviciute et al. (2017)
MS (*n* = 60) Healthy control (*n* = 43)	↑In MS	No change in abundance between patients on disease-modifying treatment and no treatment	None	Jangi et al. (2016)
MS (n = 9) Healthy control (*n* = 13)	↑In MS ↓After intervention	↑Anti-inflammatory response	2-Month Probiotic (*Lactobacillus, Bifidobacterium, Streptococcus*) by oral administration	Tankou et al. (2018)
MS in remission (n = 25) MS in relapse (*n* = 11) Healthy control (*n* = 31)	↑In MS	IgA-producing cells as a major constituent of the active immune response in MS	None	Probstel et al. (2020)
Monozygotic twin pairs (*n* = 34 pairs)	↑In untreated MS twin siblings compared to unaffected co-twin	Induced CNS-specific autoimmunity in mouse receipt post MS faecal transplantation	None	Berer et al. (2017)
Untreated MS (n = 25) Control (n = 24)	↑In MS	None	None	Cekanaviciute et al. (2018)
MS (*n* = 7) Healthy control (*n* = 8)	↑In untreated MS after vitD supplementation, compared to control and GA-treated MS	None	None	Cantarel et al. (2015)
MS (*n* = 576) Healthy control (*n* = 576)	↑In untreated MS, relapsing–remitting and progressive MS No difference within DMT groups	None	None	iMSMS Consortium. Electronic address: sergio.baranzini@ucsf.edu; iMSMS Consortium (2022)
**PD**				
PD (*n* = 51) Control (*n* = 48)	↑In PD	None	None	Li et al. (2019)
Early-stage, L-DOPA-naïve PD (*n* = 31) Control (*n* = 28)	↑In PD	Parkinsonian medications (MAO inhibitor, amantadine, dopamine agonist) did not affect taxa abundance	None	Bedarf et al. (2017)
PD (*n* = 197) Control (*n* = 103)	↑In PD	None	None	Cirstea et al. (2020)
PD (*n* = 34) Control (*n* = 34)	↑In PD	↓ Feacal SCFAs	None	Unger et al. (2016)
PD (*n* = 197) Control (*n* = 130)	↑In PD	None	None	Hill-Burns et al. (2017)
PD (*n* = 38) Control (*n* = 34)	↑In PD	↓ Feacal SCFA	None	Keshavarzian et al. (2015)
PD (*n* = 76) iRBD (*n* = 21) Control (*n* = 78)	↑In PD	None	None	Heintz-Buschart et al. (2018)
PD (*n* = 9) Control (*n* = 13)	↑In PD	None	None	Vidal-Martinez et al. (2020)
**ASD**				
ASD (*n* = 25) Control (*n* = 35)	↑In ASD (5–12-year-old)	None	None	Zurita et al. (2020)
ASD (*n* = 20) Control (*n* = 10)	↑In ASD (4–10-year-old)	None	None	De Angelis et al. (2013)
ASD (*n* = 20) Control (*n* = 28)	↑In ASD (22.4 ± 4.9 years of age)	None	None	Lee et al. (2017)
ASD (*n* = 20) Control (*n* = 20)	↑In severe ASD cases	None	none	Kang et al. (2013)
ASD (*n* = 23) Sibling control (*n* = 22) Non-sibling control (*n* = 9)	↓ In ASD (3-17-year-old)	None	None	Wang et al. (2011)
ASD (*n* = 48) Control (*n* = 48)	↓ In ASD	None	None	Zou et al. (2020)
ASD (*n* = 6) Control (*n* = 6)	Not significantly different between ASD and control (3-5-year-old)	None	None	Inoue et al. (2016)
ASD (*n* = 40) Control (*n* = 40)	Not significantly different between ASD and control (5-17-year-old)	None	None	Strati et al. (2017)
**Epilepsy**				
Children with both cerebral palsy and epilepsy (*n* = 25) Healthy control (*n* = 21)	↑ In patients aged 3–18 years	None	None	Li et al. (2019)

AD, Alzheimer’s disease; AHAD, American Heart Association Diet; ASD, autism spectrum disorders; DMT, disease modifying therapy; FCSRT, free and cued selective reminding test; GA, glatiramer acetate; MCI, mild cognitive impairment; MMKD, modified Mediterranean-ketogenic diet; MS, multiple sclerosis; MSA, multiple system atrophy; PD, Parkinson’s disease; PDD, prenatal psychological distress; iRBD, idiopathic rapid eye movement sleep behavior disorder; SCFAs, short-chain fatty acids; YKL-40, chitinase-3-like protein 1.

#### Parkinson’s disease

Postmortem studies have proposed that ɑ-synuclein inclusions, the pathological feature of PD, may be transported from the gut to the brain via the vagus nerve (Braak et al., 2006; Holmqvist et al., 2014), raising the hypothesis that PD might begin in the gut. Since then, a growing number of studies have shown alterations in diversity and richness of gut microbiota in PD patients that may trigger ɑ-synuclein misfolding or disturb function of the enteric nervous system. *A. muciniphila* has been consistently found to be increased in abundance in the guts of PD patients when compared with healthy controls (Keshavarzian et al., 2015; Unger et al., 2016; Bedarf et al., 2017; Hill-Burns et al., 2017; Li et al., 2019; Cirstea et al., 2020; Nishiwaki et al., 2020; Vidal-Martinez et al., 2020), even in those at early disease stages including L-DOPA naïve cohorts (Bedarf et al., 2017), which is thought to be due to a change of feeding behaviors including caloric restriction and fasting (Remely et al., 2015; Table 2). In addition, confounding factors such as chronic constipation and drug treatments may not be excluded when interpretating the observation of increased *A. muciniphila* abundance in faeces of PD patients. Notably, the metabolic products SCFAs have been consistently detected to be depleted in PD despite of the increased SCFAs-producing taxa *A. muciniphila*, which may be caused by other flora such as *Lachnospiraceae* and *Faecalibacterium prausnitzii* (Keshavarzian et al., 2015; Sampson et al., 2016; Unger et al., 2016). The longer colonic transit time of PD may be related to the deficiency of fecal SCFAs (Augustin et al., 2023). In addition, *A. muciniphila* has been found to be significantly more abundant in PD patients with rapid eye movement sleep behavior disorder (RBD), compared with PD patients without RBD, and correlates with nonmotor symptoms (Heintz-Buschart et al., 2018). This is particularly important, as nonmotor symptoms may appear years before the onset of motor symptoms in PD, and at present there are no reliable early biomarkers. This raises the possibility that change in the abundance or function of *A. muciniphila* may potentially serve as a suitable biomarker for the early diagnosis of PD. Longitudinal prospective studies of larger cohorts of PD patients at various stages of disease may be of value. For instance, by analyzing longitudinal data from PD patients, *A. muciniphila* has been proposed to participate in PD pathogenesis by producing hydrogen sulfide, which is a pro-inflammatory molecule harmful to the integrity of the mucus layer (Hertel et al., 2019). In a study that used three machine learning algorithms to analyze metagenomic results from 472 PD patients and 374 healthy controls, *A. muciniphila* was identified as one of the most effective bacterium among the 22 bacterial families in distinguishing between predicted PD patients and controls (Pietrucci et al., 2020). Additional studies are needed to assess the effects of anti-PD medications on the abundance of gut microbiota including *A. muciniphila*, as no significant difference in taxa abundance has been detected following administration of parkinsonian medications, including MAO inhibitors, amantadine, and dopamine agonists (Bedarf et al., 2017). Meanwhile, the potential of gut microbiota in modifying the efficacy and/or toxicity of PD medications required to be further investigated as well.

In animal studies, the elevated abundance of *A. muciniphila* has only been reported in a rotenone-treated (Dodiya et al., 2020) and in an MPTP-induced mouse models (Jeon et al., 2021), which could be associated with hyper-permeability of intestine and pro-inflammatory milieu for PD exacerbation (Dodiya et al., 2020). The elevated level of *A. muciniphila* and its related behavioral deficits and neurodegenerative damages could be suppressed by the treatment with Korean red ginseng (Jeon et al., 2021). However, the abundance of *A. muciniphila* did not increase in most mouse models of PD, including Parkinsonian A53T ɑ and MSA CNP-ɑSyn Tg mouse models (Vidal-Martinez et al., 2020), 6-OHDA-induced mouse model (Hou et al., 2021), and even decreased in a human ɑ-synuclein overexpressing mouse model (Gorecki et al., 2019).

Taking all observations together, a direct role of *A. muciniphila* and other bacteria has not yet been confirmed in PD.

#### Alzheimer’s disease and cognitive deficits

Ample evidence has shown that cognitive decline presented in patients with metabolic syndrome, and metabolic syndrome is regarded as one of the key risk factors for AD. Therefore, the prevention of metabolic syndrome may protect from developing AD. AD has been proposed as ‘type 3 diabetes’, and the anti-diabetic agents (e.g., dipeptidyl peptidase-4, also known as DPP4) has been proposed as novel therapies for AD (Cheng et al., 2020). Therefore, the correlation between *A. muciniphila* and cognitive decline was first studied in association with metabolic symptoms in rodents. For instance, high-fat diet (HFD)-fed mice has demonstrated cognitive impairments particularly in domains of contextual/spatial learning and memory, which was associated with depletion of *A. muciniphila* (Yang et al., 2019). However, a 16-week intervention with ketogenic diet could significantly increase the relative abundance of *A. muciniphila*, hence reducing the risk for neurodegeneration by improving metabolic profile in young healthy mice aged 12–14 weeks (Ma et al., 2018). In AD rodent models, a relatively lower abundance of *A. muciniphila* was found in the gut of APP/PS1 transgenic mice compared to wild-type controls, and a negative correlation between the flora and Aβ42 was observed (Harach et al., 2017). However, in another study, increased abundance of *A. muciniphila* was detected in 2-, 6-, 9-month-old APP/PS1 mice, prior to amyloidosis and microglial activation (Chen et al., 2020). Overall, beneficial effects have been observed in these mice when boosting *A. muciniphila* either by oral administration or dietary intervention (Table 1).

In human studies, *A. muciniphila* was shown to be more abundant in very mild-to-moderate AD patients compared with controls (Vogt et al., 2017), but at a similar level between individuals with mild cognitive impairment (MCI) and normal controls (Nagpal et al., 2019).

#### Autism spectrum disorder

The change in abundance of *A. muciniphila* in ASD patients remains unclear, with increased (De Angelis et al., 2013; Kang et al., 2013; Lee et al., 2017; Zurita et al., 2020) and decreased (Wang et al., 2011; Zou et al., 2020) levels demonstrated in different clinical trials, while no significant difference (Inoue et al., 2016; Strati et al., 2017) was observed in other human studies. However, small sample size has been one of the major limitations of these studies, therefore, the findings need to be confirmed in larger cohorts. Interestingly, when a gut microbiome from human donors with ASD was transplanted to germ free mice, the typical human autistic behaviors were sufficiently reproduced in mice, with an increased abundance of *A. muciniphila* and reduced abundances of Bacteroidetes and *Parabacteroides* observed in these inoculated mice (Sharon et al., 2019).

#### Epilepsy

Although the abundance of *A. muciniphila* in animal model of epilepsy is decreased (Olson et al., 2018), children with cerebral palsy and epilepsy have shown a higher abundance of compared to healthy controls (Huang et al., 2019). It was noteworthy that the abundance of *A. muciniphila* could be restored in mice by a ketogenic diet to exert protective effects against seizure (Olson et al., 2018), suggesting the potential of microbial modulation as a novel anti-seizure therapy.

### *Akkermansia muciniphila* as a potential therapeutic target for neuropsychological diseases

Given that *A. muciniphila* not only exists as a marker indicating a change in a disease state, but also plays a part in disease mechanisms, modifying abundance of the species may be of potential value in the treatment of various diseases. Although diet (Remely et al., 2015, 2016), exercise (Louis et al., 2016; Munukka et al., 2018), supplementations of *A. muciniphila* (Plovier et al., 2017; Depommier et al., 2019) or other probiotic products (Pedret et al., 2019; Tenorio-Jimenez et al., 2019), medications [e.g., antidiabetics, vancomycin or PPARɣ agonist (Wang et al., 2018; Payahoo et al., 2019; Basolo et al., 2020)] and surgeries [e.g., Roux-en-Y gastric bypass surgery, sleeve gastrectomy or duodenal/jejunal bypass surgery (Cortez et al., 2018; Sanchez-Alcoholado et al., 2019)] have all been shown to change the abundance of this species, it remains unknown whether such an increase persists and benefits individuals with neuropsychological diseases. It is noteworthy that life interventions like diets or exercises may lead to complicated microbe-microbe interactions, which are thought to be mediated by a variety of molecular and physiological mechanisms (Smid and Lacroix, 2013). Therefore, additional studies are required to figure out (i) the best method(s) for modifying *A. muciniphila* abundance; (ii) whether such a modification could exist in a long-term and be translated into neurological benefits; (iii) the potential of *A. muciniphila* as a target for the treatment of neuropsychological diseases.

Since realization of the importance of this species, studies have shown that the well-practiced interventions, such as dietary management, caloric restriction, surgical procedures, supplementation of natural products, as well as medications, all lead to an increase of *A. muciniphila* and associated beneficial effects in patients with metabolic syndromes. In addition, some other strategies have also been testified to increase the abundance of *A. muciniphila* in order to keep fitness in humans. These include: (i) a higher consumption of folate (Gurwara et al., 2019); (ii) Islamic fasting (Ozkul et al., 2019); (iii) lifestyle intervention (Guevara-Cruz et al., 2019); and (iv) a higher consumption of natural yogurt (Gonzalez et al., 2019).

#### Supplementation of *Akkermansia muciniphila*

For the APP/PS1 transgenic mouse model, supplementation of *A. muciniphila* for 6 months alleviated cognitive deficits (such as spatial learning impairments and memory deficits) as indicated by improved Y-maze test completion rates and shortened test completion time, with a significant reduction in amyloid-beta (Aβ) 40–42 levels in the cerebral cortex and in fasting blood glucose and blood lipid levels (Ou et al., 2020). In another study using an Alzheimer’s disease (AD)-like rat model with periodontitis, it was demonstrated that oral administration of this species significantly upregulated the abundance of other short-chain fatty acids (SCFAs)- or neurotransmitter-producing microbiomes and downregulated the abundance of pathogenic bacteria, resulting in alleviated cognitive impairments is association with reduced deposition of Aβ in both cerebral cortex and specific brain regions (He et al., 2022). These benefits in cognitive improvements following oral supplementation of *A. muciniphila* (or its subtype) have also been shown in mouse models with obese-induced cognitive decline, as indicated by different assessment tools such as Y-maze test (Wu et al., 2020), contextual fear-conditioning and the Barnes circular maze test (Yang et al., 2019), and novel object recognition test and spatial working memory test (Higarza et al., 2021). The mechanisms involved include that the flora restored the expression of the GluA1 and GluA2 subunits, reversed microgliosis, suppressed proinflammatory cytokines, maintained neuronal development and long-term potentiation in the hippocampus of these high-fat-diet (HFD)-fed mice (Yang et al., 2019), and the flora improved the oxidative metabolic activity in the brain by restoring the activity of the mitochondrial enzyme cytochrome C oxidase, which indicated ATP production and brain energy demand (Higarza et al., 2021). Supplementation of *A. muciniphila* was also shown to ameliorate chronic restraint stress (CRS)-induced (Ding et al., 2021) and alcohol-related (Guo et al., 2022) depressive-like behavior in mice, via enhancing intestinal barrier and maintaining gut microbiota homeostasis. In addition, *A. muciniphila* exerts beneficial effects to these depressive-like models by reducing serum LPS and neuroinflammation, normalizing the expression of depression-related genes and increasing serotonin (5-HT) levels in hippocampus (Guo et al., 2022). However, direct evidence from clinical studies is necessary to explore the therapeutic potential of *A. muciniphila* in depression and anxiety. Different strains have shown similar benefits. A treatment with *A. muciniphila* (both MucT and ATCC BAA-2869) showed similar effects of mitigation of disease progression in a mouse model of amyotrophic lateral sclerosis (ALS) by effectively increasing the nicotinamide levels in the central nervous system (CNS; Blacher et al., 2019).

A combined probiotic supplementation with other beneficial flora was also tested in various disease models (Cancello et al., 2019; Grajeda-Iglesias et al., 2021; Lee et al., 2022). In a Kcnal−/− mouse model which mimicked the associations of human KCNA1 gene variants with epilepsy and episodic ataxia, *A. muciniphila* together with *Parabacteroides* (but not alone) protected these mice against epilepsy by decreasing systemic ɣ-glutamylated ketogenic amino acids, elevating hippocampal ɣ-aminobutyric acid (GABA)/glutamate ratios and suppressing the ɣ-glutamyl transpeptidase (GGT) activity in the feaces (Olson et al., 2018).

#### Supplementation of the outer membrane protein Amuc_1100

The oral administration of Amuc_1100 was reported to ameliorate chronic unpredictable mild stress (CUMS)-induced depressive-like behavior in mice, which was in association with the improvement of CUMS-triggered down-regulation of 5-hydroxytryptamine (5-HT) and brain-derived neurotrophic factor (BDNF) and up-regulation of inflammation in hippocampus (Cheng et al., 2021).

#### Ketogenic diets and nutrient supplementations

Ketogenic diets (KD), which mimic starvation, have been shown to exert protective effects against cognitive decline. For participants with subjective memory complaints and mild cognitive impairment (MCI), the relative abundance of *A. muciniphila* was increased following modified KD in association with modulated SCFAs and improved Alzheimer’s disease (AD) biomarkers in cerebrospinal fluid (CSF), along with decreased blood glucose levels, reduced body weight and prevented decline in cognitive functions (Nagpal et al., 2019; Neth et al., 2020).

The relative abundance of *A. muciniphila* was elevated in rats under chronic mild stress following treatments with both fish oil and olive oil, however, only fish oil treatment ameliorated depressive-like behavior (Tung et al., 2019).

## Conclusion

With increasing knowledge of gut microbiota, dysbiosis of these bacteria has been recognized as a crucial player in disease mechanisms, with *A. muciniphila* as one of the overlapping bacteria involved in an increasing number of neuropsychological diseases. Although the exact mechanisms of the species are not fully understood, its change in abundance has been widely observed, showing a great potential as a biomarker at preclinical, early and progressed disease stages. It is worth considering whether the change of the bacterium is of predictive value. In neurological diseases, it is very intriguing that the abundance is down-regulated in ALS and NPDs, up-regulated in MSA, MS and PD, and inconsistent results have been observed in AD and cognitive deficits, ASD and epilepsy. It is strongly indicated that a reference point for change of this species may present to serve as a biomarker predicting disease progression from pre-clinical stage to clinical stage of one or more of these diseases, as well as to determine when interventions are required to be initiated. For instance, *A. muciniphila* holds potential as an early biomarker for the diagnosis of PD, with evidence that it is significantly related to non-motor symptoms in PD. However, due to limited studies performed, this concept needs to be confirmed with further research. In addition, a series of interventions have been proposed for *A. muciniphila* as a therapeutic target (Figure 2), suggesting that the species may also be used as an indicator for treatment response, as well as a target for therapeutic strategies. Though exciting potentials have been raised, cautions need to be taken. It remains unknown whether the abundance of the species vary considerably among individuals, and whether the level of abundance overlaps among different disease conditions. As *A. muciniphila* is sensitive to change in diet habit, exercise, nutrient supplements, medications and other medical treatments, determination of a baseline in normal cohorts remains a challenge, and requires larger-scaled studies. In addition, longitudinal studies are needed to confirm if the change of this species is transient or persistent, and whether it is suitable as a biomarker and therapeutic target.

## Author contributions

FZ performed literature search, drafted the manuscript, and prepared the figures. DW initiated, supervised, edited and revised the manuscript and finalized the work for submission. All authors contributed to the article and approved the submitted version.

## Funding

This work was funded by Important Weak Subject Construction Project of Shanghai Pudong New Area Health Commission (grant no. PWZbr2022-03).

## Conflict of interest

The authors declare that the research was conducted in the absence of any commercial or financial relationships that could be construed as a potential conflict of interest.

## Publisher’s note

All claims expressed in this article are solely those of the authors and do not necessarily represent those of their affiliated organizations, or those of the publisher, the editors and the reviewers. Any product that may be evaluated in this article, or claim that may be made by its manufacturer, is not guaranteed or endorsed by the publisher.
